# Prediction and Analysis of Tourist Management Strategy Based on the SEIR Model during the COVID-19 Period

**DOI:** 10.3390/ijerph181910548

**Published:** 2021-10-08

**Authors:** Yongdong Shi, Rongsheng Huang, Hanwen Cui

**Affiliations:** 1 School of Business, Macau University of Science and Technology, Macao 999078, China; ydshi@must.edu.mo (Y.S.); 1709853gbb30012@student.must.edu.mo (R.H.); 2 School of Computer Science, Zhuhai College of Science and Technology, Zhuhai 519041, China

**Keywords:** improved SEIR model, COVID-19, Macao, public health, economic development

## Abstract

Tourism destinations are now facing a dilemma choice of controlling the epidemic or developing the economy. This paper takes Macao, a typical international tourist city, as an example to study the strategy of tourist source control during the COVID-19 period. According to the published epidemic data of Macao, this study has established an improved SEIR (Susceptible-Exposed-Infected-Recovered) model, formulated six control strategies against the current epidemic, and used the model above to simulate the time required for all confirmed cases to recover and discharge under different strategies. By taking into consideration the gross revenue of Macao’s gambling industry from 2017 to 2019, the impact of different strategies on the economy is predicted and three control strategies are found to be feasible. This study shows that an effective way to break through the above dilemma is to design the tourist management strategy by screening the source of passengers and controlling the upper limit of capacity of destination. These findings provide a scientific basis for tourism destinations in formulating public policies. The improved SEIR model is more consistent with the actual conversion rule of patients in the current COVID-19 epidemic, and it can be applied to further public health related research.

## 1. Introduction

Controlling the epidemic or developing the economy is a difficult choice, especially for tourism destinations [1]. The COVID-19 is highly contagious, so, for the sake of public health, the number of tourists needs to be reduced and even the city shall be isolated and locked down. Many international tourist cities adopted this strategy in the early stage of the outbreak, e.g., Wuhan, Paris, Rome, New York, Macao, etc. [2,3,4,5]. When the epidemic situation was controlled and alleviated to a certain extent, these cities gradually loosened the control of tourist sources to resume work and production and develop the economy. However, this has led to a rebound in the epidemic in some areas, e.g., Zhangjiajie, most typically. Thousands of tourists are attracted by the recent performance of “Charming Xiangxi” to pack the performing hall. As a result, all the 2000 audience became close contacts [6]. An authoritative paper from Science published in 2020 predicted that the COVID-19 epidemic will always coexist with human society and there will be a small wave peak at intervals [7]. This poses a long-term and severe challenge to the choice of epidemic control and economic development [8,9,10]. Therefore, many researchers began to consider how to achieve a reasonable balance between the two [11,12,13]. Taking Macao as an example, this paper formulates six control strategies according to the actual situation of the current epidemic, and uses the improved SEIR model to simulate and predict the time required for all confirmed cases to recover and discharge under different strategies. Considering Macao’s gambling industry from 2017 to 2019, the impact of different strategies on the economy is predicted. A comparative study shows that the three control strategies are feasible. They are only permitting local residents of Macao to enter and exit Macao, permitting tourists from Guangdong Province to enter and exit Macao, and admitting 20% of the number of tourists over the past three years. These explorations and findings not only provide practical references for tourism destinations like Macao in formulating public policies, but also improve the previous SEIR model, and deepen and extend the existing theories of environmental management and public health. Therefore, dual contributions in theory and practice are expected to be achieved in this research.

Macao, located in southern China, is a special administrative region of the People’s Republic of China. As of 2019, it had a total population of 667,400 and a total land area of 32.9 km^2^. It is one of the most densely populated areas in the world. Statistics in 2018 suggest that the tertiary industry accounts for 95.8% of Macao’s total economy. The vast majority of the tertiary industry is the tourism-related gambling and hotel industry [14]. Macao’s gambling and tourism industries are in a boom, attracting an average of 35.94 million tourists from 2017 to 2019. The first case of COVID-19 infection in Macao was confirmed on 22 January 2020, and, since then, the Macao government has launched strict control over the flow of people. According to Macao’s gambling data, which is from the Statistics and Census Bureau of the Macao Special Administrative Region Government, the cumulative gross revenue of lucky gambling in Macao in 2020 decreased by 79.3%—a heavy blow to Macao’s economy—compared with that in 2019 when there was no epidemic. Throughout the epidemic, the influx of tourists, like a double-edged sword, brings huge risks to the local outbreak.

At present, COVID-19 prediction, prevention, and control are being studied by many scholars who have established models playing a positive role in regulating the pandemic [15,16,17]. Wan et al. [18] studied the data concerning the “lockdown” of Wuhan and predicted the peak number of epidemic infections in Wuhan based on the SEIR modeling method. Ivanov et al. [19] researched two SEIR models: one is described by ordinary differential equations, and the other is described by a discrete model of the first-order difference equation, predicting the peak day and the maximum number of infections. Cai et al. [20] considered the process of city management and control and simulated the epidemic situation in Wuhan. Xu et al. [21] established the differential equation of epidemic transmission based on the traditional SEIR infectious disease model and combined it with the actual situation to predict the scale of epidemic transmission. The literature reveals that the SEIR model can clearly describe the logical relationship of virus transmission and accurately predict the development trend of epidemic situations. In comparison with other models, the SEIR model has been chosen for its strengths of simplicity and involvement of lesser number of parameters in calculation for the spread of the COVID-19 pandemic in different spatial situations.

With assistance from the data of the first outbreak in Macao in early 2020 and the actual situation of Macao, this paper reorganizes the transformation process of S, E, I, and R in the SEIR model and improves the model. The epidemic situation in Macao was simulated by MATLAB, and the parameters of the model were determined. In the context of the current epidemic, this paper proposes a management and control strategy suitable for Macao Source of visitor and uses the above model to perform simulations. This strategy calculates the time required for clearing the confirmed cases (that is, all the confirmed cases are cured and discharged) with different strategies, estimates the economic loss, and compares and selects the optimal scheme, thus yielding a reasonable visitor management and control strategy for Macao to provide a scientific foundation for the new development of epidemic control in Macao—at the same time providing a reference for the development of passenger source control strategy for other tourism destinations.

## 2. Method

### 2.1. Model Selection

Since the outbreak of COVID-19, some scholars have used open data on the epidemic to establish a prediction model. The prediction methods include curve fitting, epidemic dynamics modeling, and artificial intelligence algorithms. The epidemic dynamics model, one of the most widely used models in the forecast of epidemic situations, considers the transmission speed, transmission mode, and various control measures of infectious diseases [22,23,24,25,26,27,28,29,30]. However, the parameters considered are not comprehensive, and the parameters may change dynamically in different stages of the epidemic. Therefore, the prediction effect is often unsatisfactory. Even so, these models are still of great value for their application to early warning, control decision and management, and evaluation of control [31].

The commonly used infectious disease dynamic models are the SIR model [32,33,34,35,36], SEIR model [37,38,39,40,41], MSEIR (mesoscale SEIR model) [42], SEIQCR [43], and SEIRD [44].

### 2.2. Model Construction

**Figure 1:**
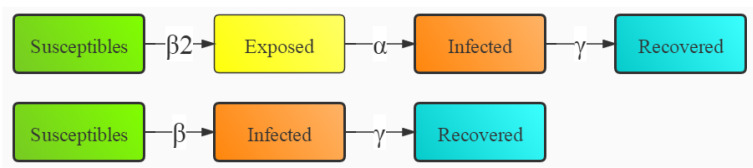
Structure diagram of the SEIR model.

The SEIR model classifies individuals as susceptible (S), exposed (E), infected (I), and recovered (R). In the article, it is constructed as shown in Figure 1 (the meaning of the symbols in Figure 1 and the equations are shown in Table 1). There are two conversion situations for healthy people: (1) conversion to latent persons and (2) conversion to patients. In the first case, the latent persons will be converted in the order of the infected, and then the recovered.

According to the conversion ideas discussed earlier, the differential equations are created. This article improves the differential equations in light of the actual situation of Macao’s COVID-19. rβIS/N represents the conversion relationship from healthy people to patients in the second conversion relationship. Therefore, the formula for calculating the latent person is moved to the formula for calculating the patient:


(1) $$\frac{\mathrm{dS}}{\mathrm{dt}}=-\frac{r\beta \mathrm{IS}}{N}-\frac{r_{2}\beta_{2}\mathrm{ES}}{N}$$

(2) $$\frac{\mathrm{dE}}{\mathrm{dt}}=-\alpha E+\frac{r_{2}\beta_{2}\mathrm{ES}}{N}$$

(3) $$\frac{\mathrm{dI}}{\mathrm{dt}}=\frac{r\beta \mathrm{IS}}{N}+\alpha E-\gamma I$$

(4) $$\frac{\mathrm{dR}}{\mathrm{dt}}=\gamma I$$

The improved differential equations above are modified into iterative form, and a MATLAB simulation is performed:


(5) $$S_{n}=S_{n-1}-\frac{{r\beta I}_{n-1}S_{n-1}}{N}-\frac{r_{2}\beta_{2}E_{n-1}S_{n-1}}{N}$$

(6) $$E_{n}=E_{n-1}-{\alpha E}_{n-1}+\frac{r_{2}\beta_{2}E_{n-1}S_{n-1}}{N}$$

(7) $$I_{n}=I_{n-1}+\frac{{r\beta I}_{n-1}S_{n-1}}{N}+{\alpha E}_{n-1}-{\gamma I}_{n-1}$$

(8) $$R_{n}=R_{n-1}+{\gamma I}_{n-1}$$

### 2.3. Data Processing

(1) Confirmation data of COVID-19

This article selects data from the first round of the COVID-19 outbreak in Macao as the research object data. The data come from the anti-COVID-19 page of the Health Bureau of the Macao Special Administrative Region Government [45]. The first case was confirmed on 22 January 2020. The 45th case was diagnosed on 8 April 2020, as recovered, and was discharged from the hospital on 19 May 2020; the 46th case was diagnosed on 26 June 2020. The two cases were diagnosed with a long time-interval and were imported cases from abroad; the 46th case was not included in the study. Therefore, there were 45 confirmed cases in the SEIR model in the article, all of whom recovered and were discharged from the hospital.

(2) Total population

The total population in this model includes data on the local population of Macao and the number of tourists visiting Macao. The data come from the Statistics and Census Bureau of the Macao Special Administrative Region Government [46]. The 2017–2019 average data are selected in the strategy research.

(3) The source of tourists

**Figure 2:**
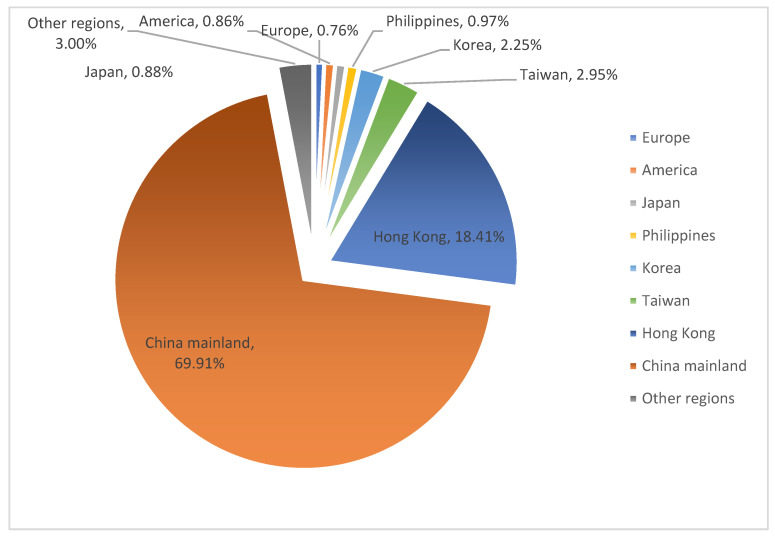
Statistics of cumulative inbound passengers based on visa issuance from 2017 to 2019.

**Figure 3:**
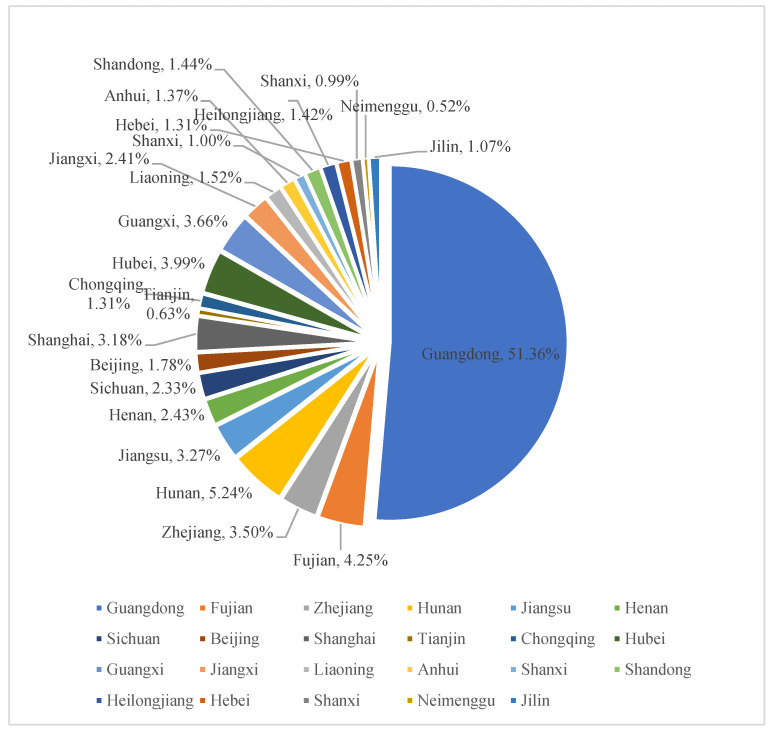
Inbound Chinese tourists by major provinces and cities from 2017 to 2019.

In line with the 2017–2019 Macao Statistical Yearbook, tourists from the mainland of China accounted for approximately 70% of Macao’s total tourist arrivals in 2017; Hong Kong was second only to China’s mainland at 18.41%; international tourist sources mainly came from East Asian countries (Japan and South Korea accounted for 0.88% and 2.25%, respectively) and Southeast Asian countries. Regarding tourists from the mainland of China, the largest number of tourists came from Guangdong Province, accounting for 51.36% of tourists. Combining the available data sources, this article assumes that all tourists entering Macao enter the casino (as shown in Figure 2 and Figure 3).

(4) Macao Casino Revenue Data

The revenue data of casinos in Macao coming from the Statistics and Census Bureau of the Macao Special Administrative Region Government [46] are used to analyze the impact of the number of tourists on the revenue of the gaming industry and appropriate casino management and control strategies.

(5) Model related data

In addition to the data above, the parameters used in the model are all derived from the Report of the WHO-China Joint Mission on Coronavirus Disease 2019 [47].

In the model, β (probability of contagious disease) is 4.8%; γ (probability of regaining health) is 99.3%; α (probability of a latent person becoming a patient) is 20%; and $\beta_{2}$ (the probability of a latent person being infected) is 1.6%.

## 3. Results

Following the SEIR model and based on China’s current main strategy to deal with the epidemic, “external defense input, internal defense rebound” as has been determined to be the normalized control, the epidemic situation in Macao is basically stabilized. China’s mainland is in a situation showing multiple irregular distributions for MATLAB simulation. Further control and management strategies of Macao casinos have yet to be studied.

**Figure 4:**
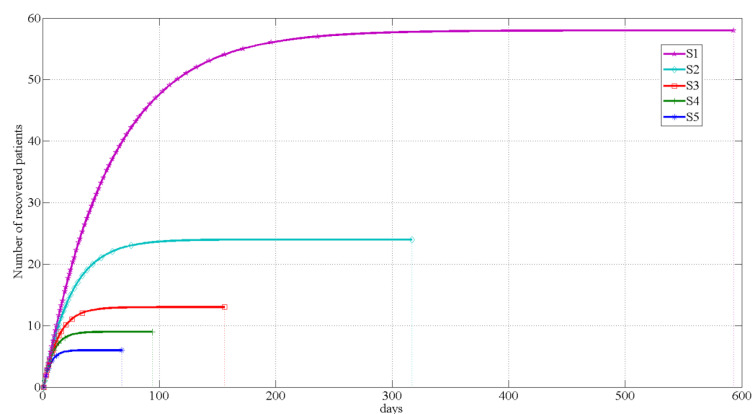
Changes in the number of patients recovered from strategies 1–5.

**Figure 5:**
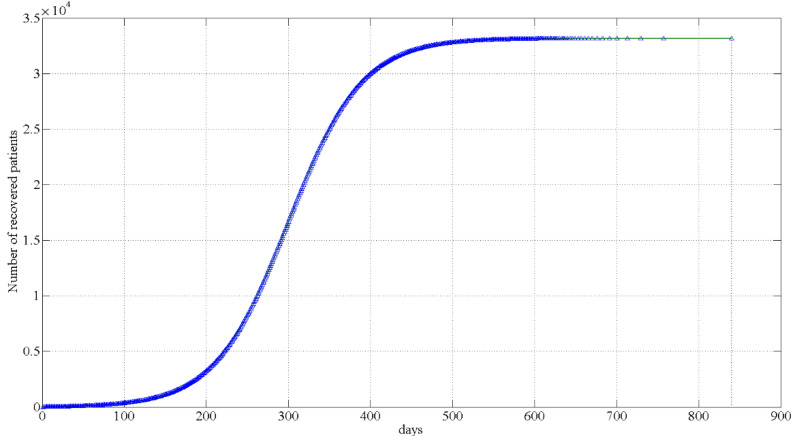
Changes in the number of recovered patients from Strategy 6.

In this paper, the following six management and control strategies are selected for MATLAB simulation, and the results are shown in Figure 4 and Figure 5.

Strategy 1: Restrict all tourists except Macao residents from entering or leaving Macao; that is, the city is closed for management. This is the most stringent control strategy.

Strategy 2: Permit tourists from Guangdong Province to enter and exit Macao, given that more than 51% of the tourists come from Guangdong Province.

The following four strategies aim to permit tourists from all over the world to enter and exit Macao while adhering to booking tours, controlling capacity, and controlling personnel proportionally; they are as follows [48]:

Strategy 3: Admit 20% of the average number of tourists over the past three years.

Strategy 4: Admit 50% of the average number of tourists over the past three years.

Strategy 5: Admit 80% of the average number of tourists over the past three years.

Strategy 6: Admit 100% of the average number of tourists over the past three years.

As shown in Figure 4 and Figure 5, the growth rate of the recovered number of strategies 1 to 5 is gradually slowing down, while the growth trend of the recovered number of strategy 6 firstly increases and then decreases, risen as an “S”-shaped curve. Owing to the number of days of recovery in strategy 6 is much greater than the previous five strategies, in order to clearly present the relationship between the number of days and the number of recovered people, strategy 6 is shown on the separate Figure 5. The simulation results suggest that, if the city remains closed for management since discovering the first case in Macao, there will be only six infected cases that will take 68 days to recover and be discharged from the hospital. If the city is not closed and only permits tourists from Guangdong Province to enter and exit Macao, the number of infection cases will be nine and will take 94 days to recover and be discharged from the hospital. If 20% of people entering Macao over the past three years are permitted to enter and exit Macao, the number of infection cases will be 13, and not until 156 days later can they be fully cured and discharged. If 50% of people entering Macao over the past three years are permitted to enter and exit Macao, the number of cases will be 24, and it will take 317 days, more than twice that of the previous control, to recover. If 80% of people entering Macao over the past three years are permitted to enter and exit Macao, the number of cases will be 58, and it will take 593 days to recover, and the length of time will gradually increase. If no control measures are taken, and all tourists are permitted to enter and leave Macao freely, the number of infection cases will increase significantly to 33,165, approximately 5% of the local population. It will take 840 days for the tourists to recover and be discharged from hospital.

**Figure 6:**
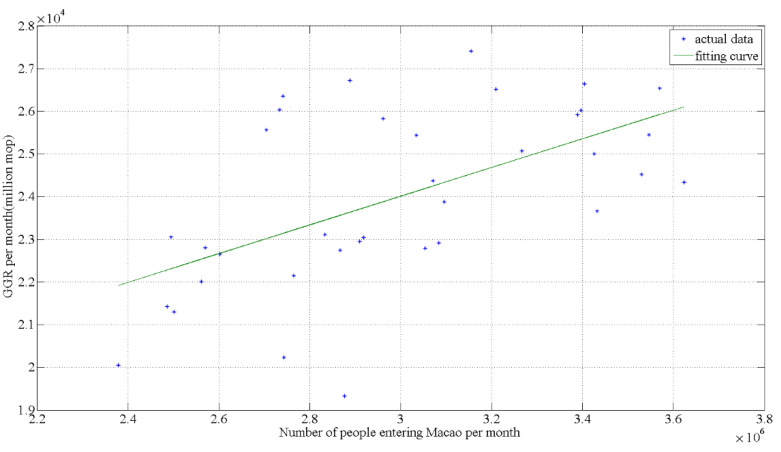
The relationship between 2017–2019 gross gaming revenue and the number of tourists in and out of Macao.

**Figure 7:**
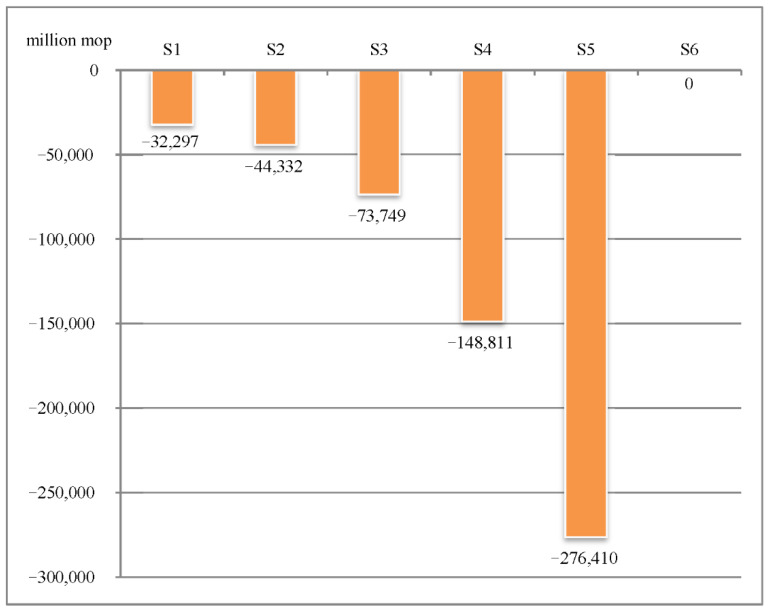
Loss of gross gaming revenue under six different management and control strategies.

Figure 6 shows the relationship between gross gaming revenue and the number of tourists in and out of Macao from 2017 to 2019. A positive correlation can be seen between the number of tourists and gross gaming revenue. Due to the control strategy mentioned previously, the gross gaming revenue is on the decline, as shown in Figure 7.

Comparing the six control strategies above, it is obvious that, although Strategy 6 has no impact on the economy at the moment, the number of patients is up to 33,165. This change is not an order of magnitude compared with other control strategies, exerting a profound impact on Macao’s gaming industry in the future. The cumulative number of confirmed cases in Strategy 1 and Strategy 2 are each in the single digits (6 and 9), and it takes 68 days and 94 days, respectively, to clear the cases. These strategies are ways to reduce economic losses, in addition to Strategy 6. In Strategies 3, 4, and 5, it takes 156, 317, and 594 days, respectively, to clear the cases, and the economic losses are 73,749 million patacas, 148,811 million patacas, and 276,410 million patacas, respectively. These three control strategies take longer than Strategies 1 and 2 and cause greater economic losses.

## 4. Conclusions

This article builds an improved SEIR model based on the epidemic data that have been published in Macao and advances six control strategies against the actual situation of the epidemic. Using the improved SEIR model for simulation, the cumulative number of confirmed cases in the first five strategies can be decreased to less than 60, but the number of confirmed cases in strategy 6 (permitting all tourists to enter and exit Macao) is 33,165, and it takes 2.3 years to clear the confirmed cases. Therefore, without control measures, more damage will be done to Macao. If the control is carried out according to the different percentages of average tourists from 2017 to 2019, it will take more than 100 days for the confirmed cases to be cleared. Combining the SEIR simulation results and the loss of gross gaming revenue, Strategy 1 (only permitting local residents of Macao to enter and exit Macao), Strategy 2 (permitting tourists from Guangdong Province to enter and exit Macao), and Strategy 3 (admitting 20% of the number of tourists over the past three years) are feasible.

The results above show that, during the period of COVID-19, to design the tourist management strategy by screening the source of passengers and controlling the upper limit of capacity of destination is an effective way to break through the dilemma of controlling the epidemic or developing the economy. If tourism destinations are merely concerned about the immediate economic benefits without taking any epidemic control, a large number of people will be infected, and the economy will be seriously wrecked. On the other hand, it can only be a short-term expedient to tighten the source of tourists and adopt isolation and closure. If it goes on like this in the long run, its economic cost will be too huge to be borne. Therefore, to formulate effective management and control strategies, tourism destinations need to implement real-name registration to identify the main source of tourists. Moreover, it is necessary to determine the capacity upper limit of the scenic spot, and master the basic relationship between the number of tourists, source, and economic income in the past several years. Then, with reference to the ideas and methods of this research, a more reasonable source management and control strategy can be produced to fit for their own development. These results are consistent with the findings of Moosa et al. [49], as a response to the controversial issue in previous literature. Although the methods and techniques in our study are different from theirs, the final control strategies both led to restricting international travel to prevent the spread of epidemic. In addition, this study fills a gap in the existing literature. Due to the unavailability of data, previous relevant studies failed to include Macao as a research object [50].

This paper has improved the previous SEIR model [51,52] in that the conversion process of the case state is divided into two: one is the conversion from healthy people to latent persons, and then in the order of the infected and the recovered; the other is the direct conversion from healthy people to patients, and then to the recovered. The differential equation in other studies is also further improved by moving the formula of calculating latent persons into the formula of calculating the infected persons in the conversion process. This improvement is more in line with the reality of COVID-19 in Macao and closer to the current situation of large-scale vaccination worldwide. Mass vaccination can effectively reduce the hospitalization rate, severe disease rate, and mortality. Although there has been an increasing severity of Delta variant recently, and there have been breakthrough cases of infection after vaccination, these cases account for only a small proportion of people among those 3 billion who have been vaccinated in the world. Therefore, the research results of this paper can be used as a reference for follow-up researchers to apply the SEIR model to public health related research.

The improved SEIR model in this paper can better predict the development trend of COVID-19 in Macao. It can be used to predict the outcomes of different epidemic control strategies and provide scientific guidance for the COVID-19 control in Macao, considering economic losses. The improved SEIR model can be applied to other tourism destinations with epidemic diseases to help them cope with the epidemic more effectively, which is also the direction of our follow-up research. The limitations of this study are that, because the spread process of the COVID-19 is very complex, we cannot fully examine every factor affecting the spread of the epidemic, such as the different infection rates of different age and gender groups, the different infection rates between healthy people and those who have underlying diseases, and the influence of different regional population density on the spread and the impacts brought about by the adequacy or shortage of medical resources and different quarantine measures. However, it is difficult for us to take into account all these factors in the model, which has a certain impact on the simulation results of the SEIR model. If relevant data are available, we can add them in future research to improve the simulation accuracy of the SEIR model.

## Figures and Tables

**Table 1:** Symbolic meaning of the SEIR model.

Symbol	Meaning	Symbol	Meaning
N	Population size	β	Probability of spreading disease
S	Susceptible	γ	Probability of recovery
I	Infected	α	Probability of the exposed turning into the infected
E	Exposed	$\beta_{2}$	Transmission probability of the exposed
r	Amount of daily contact with the infected	$r_{2}$	Amount of daily contact with the exposed
R	Recovered		

## Data Availability

Data available in a publicly accessible repository: (1) Health Bureau of the Macao Special Administrative Region Government, https://www.ssm.gov.mo/; (2) Statistics and Census Bureau of the Macao Special Administrative Region Government, https://www.dsec.gov.mo/; (3) Report of the WHO-China Joint Mission on Coronavirus Disease 2019, https://www.who.int/.

## References

[1] Duro J.A., Perez-Laborda A., Turrion-Prats J., Fernández-Fernández M. (2021). COVID-19 and tourism vulnerability. Tour. Manag. Perspect..

[2] Lau H., Khosrawipour V., Kocbach P., Mikolajczyk A., Schubert J., Bania J., Khosrawipour T. (2020). The positive impact of lockdown in Wuhan on containing the COVID-19 out-break in China. J. Travel Med..

[3] Di L., Pullano G., Sabbatini C., Boëlle P.Y., Colizza V. (2020). Impact of lockdown on COVID-19 epidemic in le-de-France and possible exit strategies. BMC Med..

[4] Tarra S., Mazzocchi G., Marino D. (2021). Food system resilience during COVID-19 Pandemic: The Case of roman solidarity purchasing groups. Agriculture.

[5] Ambrosio B., Aziz-Alaoui M.A. (2020). On a coupled time-dependent sir models fitting with New York and New-Jersey states COVID-19 data. Biology.

[6] The Epidemic in Nanjing Spread across Provinces, Loopholes in Epidemic Prevention Have Been Exposed at Airports and Scenic Spots. http://www.chinanews.com/sh/2021/07-30/9532057.shtml.

[7] Kissler M., Tedijanto C., Goldstein E., Grad Y.H., Lipsitch M. (2020). Projecting the transmission dynamics of SARS-CoV-2 through the postpandemic period. Science.

[8] Zhang D., Hu M., Ji Q. (2020). Financial markets under the global pandemic of COVID-19. Financ. Res. Lett..

[9] Haroon O., Ali M., Khan A., Khattak M.A., Rizvi S.A.R. (2021). Financial market risks during the COVID-19 Pandemic. Emerg. Mark. Finance Trade.

[10] Liu K. (2021). COVID-19 and the Chinese economy: Impacts, policy responses and implications. Int. Rev. Appl. Econ..

[11] Goodell J.W. (2020). COVID-19 and finance: Agendas for future research. Financ. Res. Lett..

[12] Akhtaruzzaman M., Boubaker S., Lucey B.M., Sensoy A. (2021). Is gold a hedge or a safe-haven asset in the COVID–19 crisis?. Econ. Model..

[13] Albulescu C.T. (2021). COVID-19 and the United States financial markets’ volatility. Financ. Res. Lett..

[14] Bai Y., Li Y. (2020). Macau’s new coronary pneumonia epidemic emergency mechanism and emergency finance enlightenment. Sub. Natl. Fisc. Res..

[15] Wang C., Pan R., Wan X., Tan Y., Xu L., Ho C.S., Ho R.C. (2020). immediate psychological responses and associated factors during the initial stage of the 2019 Coronavirus Disease (COVID-19) epidemic among the general population in China. Int. J. Environ. Res. Public Health.

[16] Mazza C., Ricci E., Biondi S., Colasanti M., Ferracuti S., Napoli C., Roma P. (2020). A nationwide survey of psychological distress among Italian people during the COVID-19 pandemic: Immediate psychological responses and associated factors. Int. J. Environ. Res. Public Health.

[17] Peng X., Tang X., Chen Y., Zhang J.H. (2021). Ranking the healthcare resource factors for public satisfaction with health system in China—based on the grey relational analysis models. Int. J. Environ. Res. Public Health.

[18] Wan K., Chen J., Lu C., Dong L., Wu Z., Zhang L. (2020). When will the battle against novel coronavirus end in Wuhan: A SEIR modeling analysis. J. Glob. Health.

[19] Ivanov S.V., Leonenko V.N. (2017). Prediction of influenza peaks in Russian cities: Comparing the accuracy of two SEIR models. Math. Biosci. Eng..

[20] Cai J., Jia H., Wang K. (2020). Prediction of development trend of COVID-19 in Wuhan based on SEIR model. Shandong Med. J..

[21] Xu J., Yang M. (2020). Prediction of the scale of infection of novel coronavirus pneumonia based on SEIR model. Sci. Technol. Innov..

[22] Xiang Y., Jia Y., Chen L., Guo L., Shu B., Long E. (2021). COVID-19 epidemic prediction and the impact of public health interventions: A review of COVID-19 epidemic models. Infect. Dis. Model..

[23] Parino F., Zino L., Porfiri M., Rizzo A. (2021). Modelling and predicting the effect of social distancing and travel restrictions on COVID-19 spreading. J. R. Soc. Interface.

[24] Borah M.J., Hazarika B., Panda S.K., Nieto J.J. (2020). Examining the correlation between the weather conditions and COVID-19 pandemic in India: A mathematical evidence. Results Phys..

[25] Saha S., Samanta G.P. (2021). Modelling the role of optimal social distancing on disease prevalence of COVID-19 epidemic. Int. J. Dyn. Control..

[26] Alberti T., Faranda D. (2020). On the uncertainty of real-time predictions of epidemic growths: A COVID-19 case study for China and Italy. Commun. Nonlinear Sci. Numer. Simul..

[27] Wei Y., Wang J., Song W., Xiu C., Ma L., Pei T. (2021). Spread of COVID-19 in China: Analysis from a city-based epidemic and mobility model. Cities.

[28] Saha S., Samanta G.P., Nieto J.J. (2020). Epidemic model of COVID-19 outbreak by inducing behavioural response in population. Nonlinear Dyn..

[29] Giamberardino P., Iacoviello D. (2021). Evaluation of the effect of different policies in the containment of epidemic spreads for the COVID-19 case. Biomed. Signal. Process. Control..

[30] Péni T., Csutak B., Szederkényi G., Röst G. (2020). Nonlinear model predictive control with logic constraints for COVID-19 manage-ment. Nonlinear Dyn..

[31] Huang L., Wei Y., Shen S., Zhu C., Chen F. (2020). Evaluation of predictive models for novel coronavirus pneumonia. Chin. J. Health Stat..

[32] Liu X. (2021). A simple, SIR-like but individual-based epidemic model: Application in comparison of COVID-19 in New York City and Wuhan. Results Phys..

[33] Taghizadeh L., Karimi A., Heitzinger C. (2020). Uncertainty quantification in epidemiological models for the COVID-19 pandemic. Comput. Biol. Med..

[34] Senel I., Ozdinc M., Ozturkcan S. (2020). SPE approach for robust estimation of SIR Model with limited and noisy data: The case for COVID-19. Disaster Med. Public Health Prep..

[35] Zhang Y. (2020). Study of COVID-19 Based on SIR Model.

[36] Rocchi E., Peluso S., Sisti D., Carletti M. (2020). A possible scenario for the COVID-19 epidemic, based on the SI(R) model. SN Compr. Clin. Med..

[37] Lacitignola D., Saccomandi G. (2021). Managing awareness can avoid hysteresis in disease spread: An application to coronavirus COVID-19. Chaos Solitons Fractals.

[38] Postavaru O., Anton S., Toma A. (2021). COVID-19 pandemic and chaos theory. Math. Comput. Simul..

[39] Silva P., Batista P., Lima H., Alves M.A., Guimarães F.G., Silva R.C. (2020). COVID-ABS: An agent-based model of COVID-19 epidemic to simulate health and eco-nomic effects of social distancing interventions. Chaos Solitons Fractals.

[40] Zhu Y., Huang B., Wang Z., Ju J., Zhu L. (2020). Analysis of the isolation measure on the control model of COVID-J. Wuhan Univ. (Nat. Sci. Ed.).

[41] Dolbeault J., Turinici G. (2020). Heterogeneous social interactions and the COVID-19 lockdown outcome in a multi-group SEIR model. Math. Model. Nat. Phenom..

[42] Chen X., Zhang A., Wang H., Gallaher A., Zhu X. (2021). Compliance and containment in social distancing: Mathematical modeling of COVID-19 across townships. Int. J. Geogr. Inf. Sci..

[43] Xu L., Liu W., Liu Y., Li M., Luo L., Ou C. (2020). Construction of SEIQCR epidemic model and its application in the evaluation of public health interventions on COVID-19 in Guangzhou. J. Shandong Univ. (Health Sci.).

[44] Maugeri A., Barchitta M., Battiato S., Agodi A. (2020). Estimation of unreported novel coronavirus (SARS-CoV-2) infections from re-ported deaths: A susceptible–exposed–infectious–recovered–dead model. J. Clin. Med..

[45] The Research Object Data of First Round of the Outbreak of COVID-19 in Macao. https://www.ssm.gov.mo/apps1/.PreventCOVID-19/ch.aspx#clg17458.

[46] The Revenue Data of Casinos in Macao. https://www.dsec.gov.mo/zh-MO/.

[47] Report of the WHO-China Joint Mission on Coronavirus Disease 2019 (COVID-19). https://www.who.int/docs/default-source/coronaviruse/who-china-joint-mission-on-COVID-19-final-report.pdf.

[48] Shi P., Lu M. (2020). Guaranteeing tourism health in the context of regular epidemic prevention and control—New perspectives and new innovations in addressing the impact of COVID-19 on tourism. J. Xinjiang Normal University (Philos. Soc. Sci.).

[49] Moosa I.A., Khatatbeh I.N. (2020). International tourist arrivals as a determinant of the severity of COVID-19: International cross-sectional evidence. J. Policy Res. Tour. Leis. Events.

[50] Ning J., Chu Y., Liu X., Zhang D., Zhang J., Li W., Zhang H. (2021). Spatio-temporal characteristics and control strategies in the early period of COVID-19 spread: A case study of the mainland China. Environ. Sci. Pollut. Res..

[51] Linka K., Peirlinck M., Kuhl E. (2020). The reproduction number of COVID-19 and its correlation with public health interventions. Comput. Mech..

[52] Feng S., Feng Z.B., Ling C., Chang C., Feng Z. (2021). Prediction of the COVID-19 epidemic trends based on SEIR and AI models. PLoS ONE.

